# Comparative Analyses of Reproductive Structures in Harvestmen (Opiliones) Reveal Multiple Transitions from Courtship to Precopulatory Antagonism

**DOI:** 10.1371/journal.pone.0066767

**Published:** 2013-06-10

**Authors:** Mercedes M. Burns, Marshal Hedin, Jeffrey W. Shultz

**Affiliations:** 1 Department of Entomology, University of Maryland, College Park, Maryland, United States of America; 2 Department of Biology, San Diego State University, San Diego, California, United States of America; Estacion Experimental de Zonas Áridas (CSIC), Spain

## Abstract

Explaining the rapid, species-specific diversification of reproductive structures and behaviors is a long-standing goal of evolutionary biology, with recent research tending to attribute reproductive phenotypes to the evolutionary mechanisms of female mate choice or intersexual conflict. Progress in understanding these and other possible mechanisms depends, in part, on reconstructing the direction, frequency and relative timing of phenotypic evolution of male and female structures in species-rich clades. Here we examine evolution of reproductive structures in the leiobunine harvestmen or “daddy long-legs” of eastern North America, a monophyletic group that includes species in which males court females using nuptial gifts and other species that are equipped for apparent precopulatory antagonism (i.e., males with long, hardened penes and females with sclerotized pregenital barriers). We used parsimony- and Bayesian likelihood-based analyses to reconstruct character evolution in categorical reproductive traits and found that losses of ancestral gift-bearing penile sacs are strongly associated with gains of female pregenital barriers. In most cases, both events occur on the same internal branch of the phylogeny. These coevolutionary changes occurred at least four times, resulting in clade-specific designs in the penis and pregenital barrier. The discovery of convergent origins and/or enhancements of apparent precopulatory antagonism among closely related species offers an unusual opportunity to investigate how major changes in reproductive morphology have occurred. We propose new hypotheses that attribute these enhancements to changes in ecology or life history that reduce the duration of breeding seasons, an association that is consistent with female choice, sexual conflict, and/or an alternative evolutionary mechanism.

## Introduction

Structures and behaviors associated with animal reproduction typically differ even among closely related species, although stability within a species tends to be maintained [1]–[2]. However, the mechanisms responsible for producing this widespread pattern remain uncertain even after 150 years of dedicated research by evolutionary biologists. Some workers have proposed a role for natural selection in reproductive diversification, either directly via lock-and-key mechanisms [3]–[4] or indirectly via pleiotropy [5], but there is little evidence for these processes in most systems that have been studied [2]. A number of sexual selection mechanisms have also gained purchase in functional and evolutionary reproductive diversification paradigms. These include the perennial female choice—both obvious and cryptic [6]–[11], and the more-recent intersexual conflict [12]–[18] and sperm competition mechanisms [19]–[21].

Which evolutionary processes lead to the diversification of reproductive structures? An evolutionary question of this magnitude requires diverse perspectives and approaches that include theory, experimentation and development of model organisms, all of which are fairly well represented in the recent literature. However, the phylogenetic comparative approach—wherein the direction, frequency and evolutionary context of specific evolutionary transformations are explored within species-rich clades—has been used less frequently to understand mating system diversity. This is despite the demonstrated value of this approach for understanding evolutionary patterns in other aspects of organismal biology, such as feeding and geographic distribution [22]–[23]. The recent paucity of such studies as applied to reproductive structures probably reflects the difficulty in targeting large clades that have undergone relevant evolutionary changes and for which a well-resolved phylogeny is available. In addition, these approaches can suffer from uncertainties inherent in all historical reconstructions [24]–[25]. Still, the phylogeny-based historical approach aids in the description and explanation of diversification that has occurred in natural systems at different evolutionary time scales. These perspectives are not available with single species studies or in comparative analyses that use phylogeny solely for the removal of statistical non-independence due to species relatedness.

**Figure 1:**
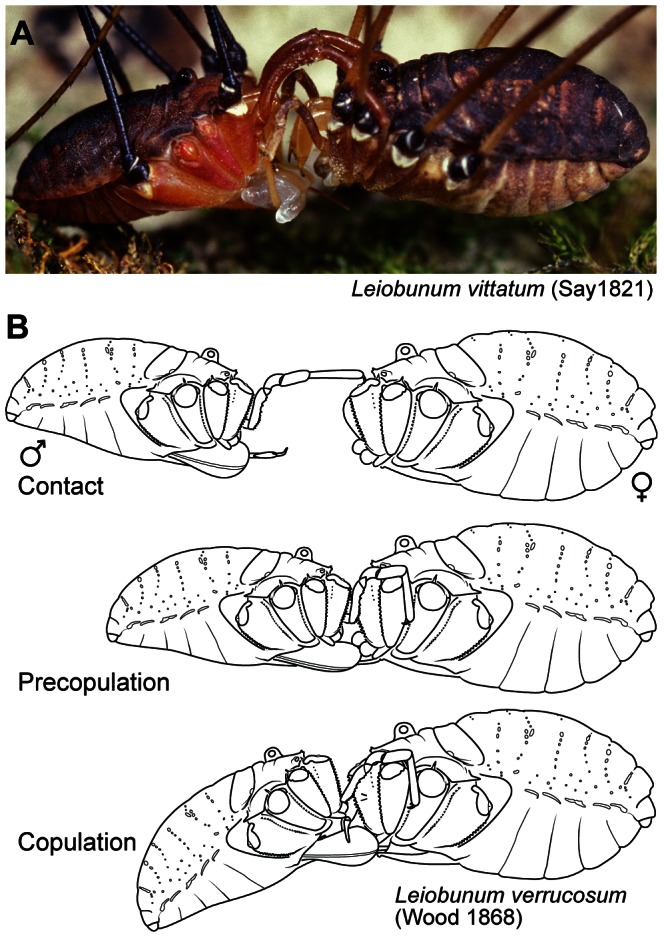
Mating behavior and morphology in leiobunine harvestmen. (A) Precopulatory behavior in *Leiobunum vittatum*. Male on left, female on right. Photograph courtesy of Joe Warfel (Eighth-Eye Photography). (B) Major phases in mating in *Leiobunum verrucosum* (semi-diagrammatic, legs not included for clarity).

**Figure 2:**
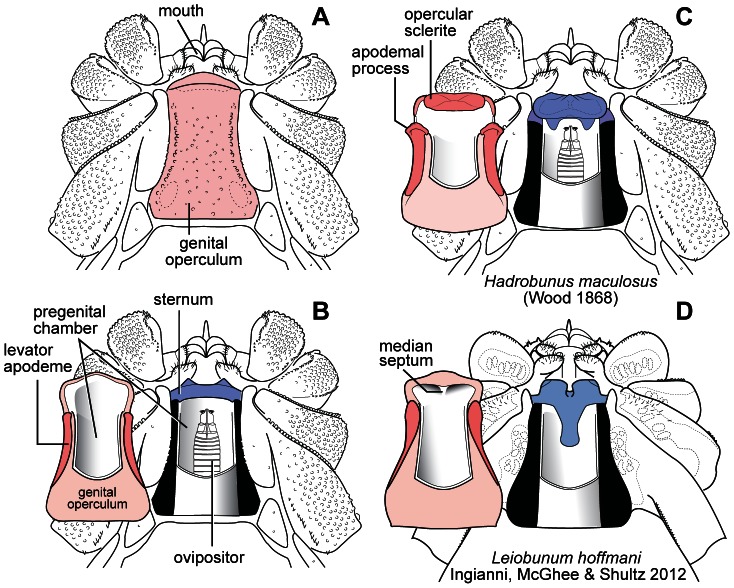
Female genital morphology in leiobunine harvestmen. (A) Ventral surface of generalized female showing relative positions of the feeding apparatus and pregenital opening. (B) As in A, but with genital operculum removed and flipped to show the inner structures of a simple (primitive) operculum and sternum, not modified into a pregenital barrier. (C) Ventral surface of *Hadrobunus maculosus* from same perspective as B, showing pregenital barrier (see also Fig. 3). The large sclerotized sternum engages the opercular sclerite anteriorly and apodemal processes posteriorly. (D) Ventral surface of *Leiobunum hoffmani* from same perspective as B and C, showing pregenital barrier (see also Fig. 3). The anterior median notch in the sclerotized sternum engages a sclerotized median septum on the genital operculum; the posterior margin of the sternum abuts the anterior margin of the levator apodeme (based on [54]). In both C and D, a barrier is formed by a sclerotized sternum wedged between anterior and posterior elements of the genital operculum.

**Figure 3:**
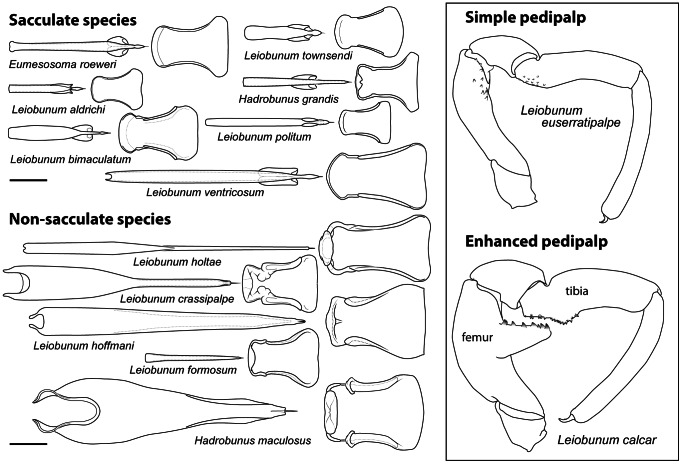
Structures from representative sacculate and non-sacculate species of leiobunine harvestmen. Penes are depicted from a dorsal view. The genital opercula are shown from the inner (dorsal) perspective (compare with Figs. 2B-D). All penes and opercula (right box) are drawn to the same scale; bar = 1 mm. The pedipalps are from male *Leiobunum euserratipalpe* and *L.calcar* [54]. Simple male pedipalps are roughly similar in shape and relative size to those of females. The enhanced male pedipalps (left box) depicted have femoral apophyses which are used in concert with the base of the tibia to clamp the trochanter of the female's first pair of legs during mating. See Fig. 1A for a different form of enhanced male pedipalp, in which the overall length of the pedipalps is sexually dimorphic (longer in males relative to females).

Here we examine evolutionary patterns in the reproductive morphology of the leiobunine harvestmen or "daddy longlegs" of eastern North America. The group encompasses three genera—*Leiobunum*, *Eumesosoma* and *Hadrobunus*—with about 35 described species and 12 known-but-undescribed species. The taxonomic nomenclature of the group is currently in flux but recent molecular systematic analyses have revealed the monophyly of the group and its basic phylogenetic structure [26]–[27]. The reproductive morphology of the clade is diverse, but much of this diversity can be captured by three binary, categorical variables, that is, a penis with or without nuptial gift sacs, a female pregenital apparatus with or without a sclerotized barrier, and male pedipalps similar in size and shape to those of females or mechanically enhanced for clasping (Figs. 1–3). There is an apparent tendency for these traits to occur in two morphology-based syndromes, one that is consistent with a mating system in which females choose males based on a precopulatory nuptial gift (courtship) and one in which precopulatory contact involves large or prolonged mechanical forces with more limited exchange of nuptial gifts (precopulatory antagonism). The goals for this study are thus 1) to reconstruct the direction and frequency of trait evolution, 2) to determine whether the two syndromes are real and reflected in correlated evolution of traits and 3) to determine if morphological change in a focal trait tends to precede or follow change in another trait.

Background: Mating and Reproductive Morphology in Leiobunine Harvestmen.

In general, mating behavior in the leiobunine harvestmen is broadly divided into precopulatory and copulatory phases (Fig. 1). During the precopulatory phase the male uses his pedipalps to grasp the female behind the base of her second leg pair (coxa II); the male and female are positioned face-to-face with the long axes of their bodies in rough alignment [28]. The penis is usually everted during this phase and its tip may contact the female pregenital opening, but it does not penetrate the pregenital chamber (Fig. 2). The male offers a nuptial gift from accessory glands positioned near the opening to his pregenital chamber. The copulatory phase is characterized by penetration of the penis into the pregenital chamber and a change in body position in which the male assumes a more "face up" orientation. Insemination occurs within the pregenital chamber. These features of mating appear to be universal among the leiobunines of eastern North American, but details of reproductive morphology and mating behavior differ among species.

Species can be broadly divided into two categories: sacculate and non-sacculate (Fig. 3). In sacculate species, the penis has a bilateral pair of subterminal cuticular sacs that contain a secretion derived from accessory glands [26]. When a male encounters a receptive female, he clasps her with his pedipalps and inserts the penis into the female's mouth. The penis is rapidly withdrawn and its distal end is placed at the opening to the female's pregenital chamber. The primary nuptial gift is followed by a secondary gift issued directly from the accessory glands. The female spends a variable amount of time (a few seconds to a few minutes) appearing to feed on the secretion. Although the chemical profile of the secretion and its potential effects on female fecundity are unknown, the female's active reception of the material and the apparent ubiquity of its transmission indicate that the label of nuptial gift is warranted [29]. The copulatory phase of mating begins when the female opens the genital operculum and the male re-orients into the copulatory posture (Fig. 1). Females reject males by running away or adopting a face-down orientation [28].

Many non-sacculate species begin mating in a similar way, but little, if any, primary nuptial gift is transferred. In some species, the male pedipalps are modified for strongly clasping the female (Fig. 3). The sterno-opercular mechanisms of females are usually sclerotized and appear to serve as reinforced pregenital barriers (Figs. 2C, 2D). The duration of the precopulatory phase varies considerably and can last for up to an hour. In some species, the pair maintains their precopulatory posture for long periods with brief intervals of struggling in which the male makes attempts at forcefully penetrating the female's pregenital chamber. We have not observed enough interactions to determine how often these encounters end in copulation.

## Materials and Methods

### Taxon Sample

**Figure 4:**
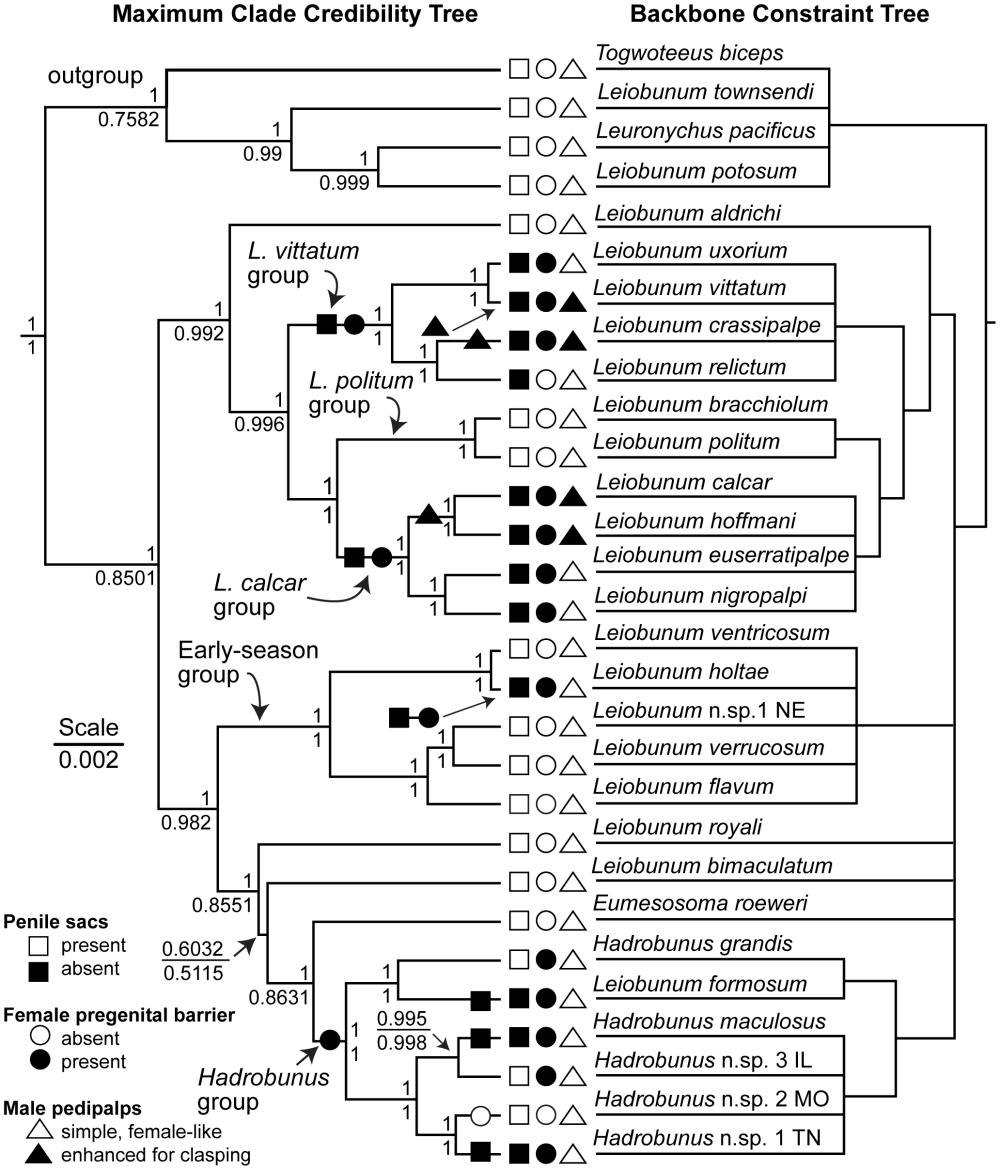
Phylogenetic hypotheses and distribution of reproductive characters. The maximum clade credibility Bayesian tree (left) was assembled using the TreeAnnotator program [31], visualized with FigTree v1.3.1 [73], and depicts relationships recovered in BEAST v1.7.1 [31] for trees that passed the backbone constraint tree (right). Values above branches indicate the posterior probabilities per node for filtered trees (n = 431). Values below braches are the posterior probabilities of the maximum clade credibility tree for a subset of 1000 random trees resampled from the original posterior probability distribution. Scale is in substitutions per site for the filtered subset maximum clade credibility tree. The most parsimonious distribution of reproductive characters (assuming no parallel gains in penile sacs) is mapped to the maximum clade credibility tree. Geographic codes are given for undescribed species: IL = Illinois, MO = Missouri, NE = Nebraska, TN = Tennessee. The backbone constraint tree (right) depicts relationships that were well supported (>95% posterior probability) in the [26] tree and that were used to generate sets of trees for the present study.

Analyses were conducted using 25 species from the eastern North American clade of leiobunine harvestmen, and four outgroup species from a closely related clade occurring in Mexico and the western United States [27]. The sample included all genera and all but six described species from the eastern clade plus four undescribed species. Because discrete genital morphology does not vary within species and monophyly of species groups is well supported [26] (Fig. 4), we conducted our analyses using one population (i.e., one tip) from each multiply represented species examined by [26] in both the phylogenetic and morphological analyses (Fig. 4). Table S1 in the supplementary materials includes additional details regarding taxon sampling for molecular and morphological assignment.

### Phylogenetic Trees

All analyses were conducted using as a template the phylogenetic tree recovered by Bayesian analysis of mitochondrial and nuclear genes in [26]. However, because the branch lengths of the original topology reflect rates of molecular rather than morphological evolution, we used the same molecular data (see Table S1 for GenBank accession numbers) to generate a set of ultrametric trees in which internodal lengths reflect time and lengths of all root-to-tip pathways were equal [30]. Ultrametric trees were constructed using BEAST v1.7.1 [31] assuming a Yule speciation process prior. The data matrix was divided into three partitions—mitochondrial DNA, 28 S rDNA and elongation factor 1-α—analyzed simultaneously using separate GTR+I+Γ models. Ultrametric branch lengths were calculated using unlinked and uncorrelated log-normal relaxed clocks separated by partition [32]. Two independent tree-searching analyses each ran for 100 million iterations, where one configuration was sampled per 1000 generations with the default 10% burn-in (Data deposited in the Dryad on-line repository: (http://dx.doi.org/10.5061/dryad.79d15).

The program TRACER v1.5 [33] was used to ensure that effective sample sizes of the posterior distribution were greater than 1000 for each independent analysis. To achieve a more conservative burn-in of 30%, we discarded an additional 20% of sampled trees using LogCombiner v1.7.1 [31]. The posterior distributions of the two analyses were pooled to yield 1000 trees. Multiply represented taxa were pruned to one population per species (see Table S1 for localities) by list-applying (command ‘lapply’) the “drop.tip” function to the entire set of trees in the ape package [34] available through the R statistical computing language [35]. To ensure consistency with the branching pattern of the original Bayesian tree [26], the posterior distribution was filtered using a rooted backbone constraint tree (Fig. 4) in PAUP* v4.0b [36] which preserved well-supported clades (i.e., posterior probabilities >0.95) while allowing for variation in the placement of poorly supported clades and species. This resulted in a distribution of 431 trees that was used in all analyses of character evolution.

### Evolution of the Penis and Male Pedipalps

**Figure 5:**
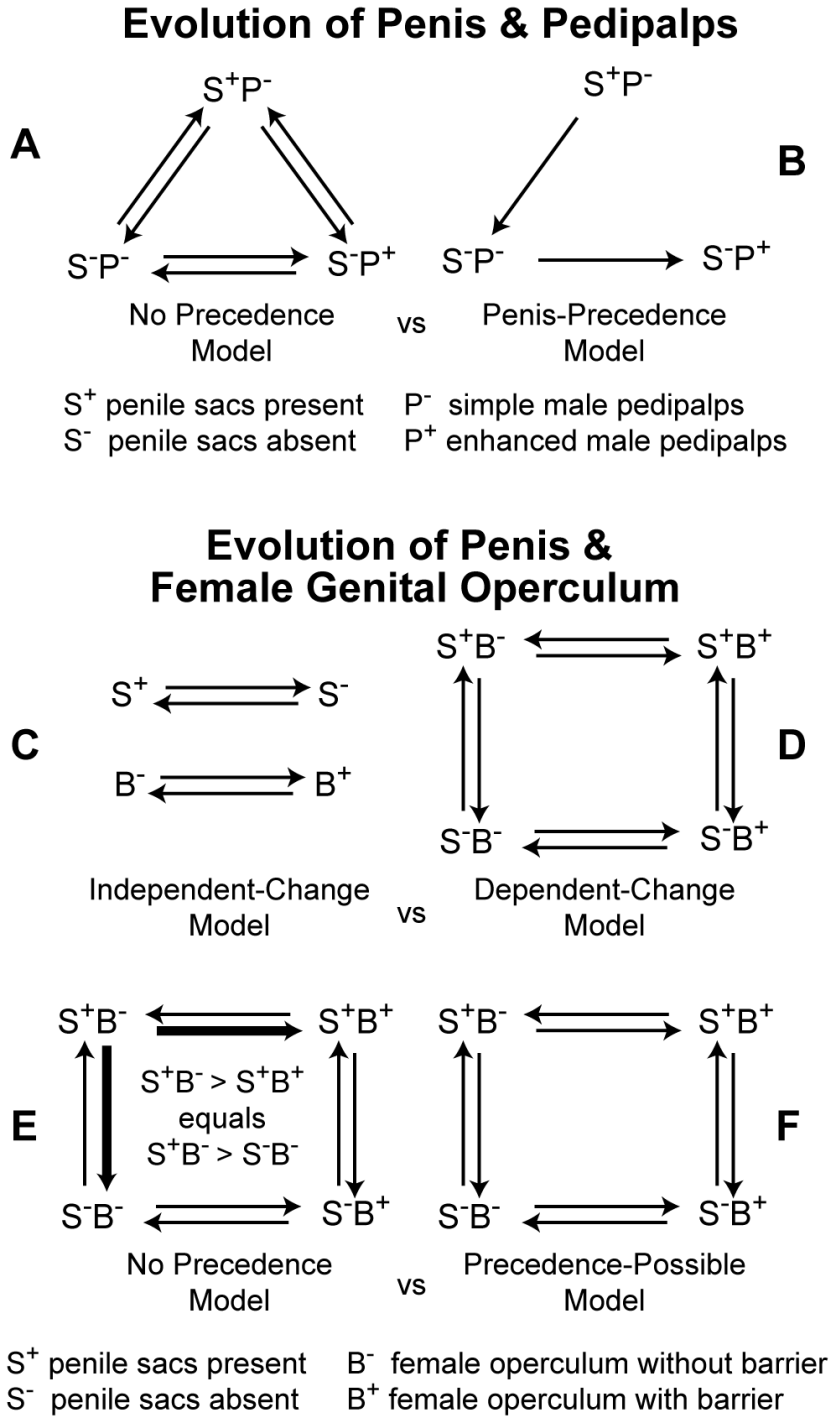
Transition models used to test hypotheses for the evolution of reproductive characters with Bayesian analysis. (A) No-precedence model of male morphological evolution versus (B) Penis precedence model, where male morphological transitions are limited to sacculate (S^+^) to nonsacculate (S^−^) penis and simple pedipalps (P^−^) to enhanced pedipalps (P^+^). The root of A was treated as fixed to S^+^P^−^ (Table 1, row 3) or determined empirically (Table 1, row 1). (C) Independent and (D) dependent models of discrete male and female reproductive morphology. Here, the female pregenital barrier is coded as present (B^+^) or absent (B^−^). Both models allow for all possible character transitions. (E) No precedence model was compared to dependent model (F), where character precedence is possible. In this model, penile sac loss (S^+^B^−^ →S^−^B^−^) and barrier acquisition (S^+^B^−^ →S^+^B^+^) are constrained to have equal rates of evolution.

Males of each species were assigned one of three combinations of penile-sac (S) and pedipalpal (P) features. Species with bilateral penile cuticular sacs that convey a nuptial gift [37] and simple “female-like” pedipalps were coded as S^+^P^−^; species that lack penile sacs and have simple pedipalps were coded as S^−^P^−^; and species that lack sacs but have pedipalps heavily modified for clasping (Fig. 3) were coded as S^−^P^+^ (Figs. 5A, 5B). States were determined for all species by original observations of anatomy. No species is known to have both penile sacs and modified pedipalps, so this combination of traits was not coded. That this combination is unobserved gives strength to our alternative model of male reproductive evolution, so we chose to ignore it, although alternative approaches might include the combination [38].

The ancestral male morphology was determined using parsimony reconstructions with Mesquite v. 2.75 [39] and with BayesTraits Multistate [40]–[41]. The latter was accomplished by comparing marginal likelihoods of two models: a 6-rate model in which all transitions between character states were possible, and a model that differs only in that state 0 (i.e., no penile sacs, simple pedipalps) was assigned to the root. As these models are not nested, they were compared using Bayes factors [42].

To assess the direction of change in male morphology, two potential models of male character evolution were compared: the 6-rate model representing the possibility for transitions between all three character states (Fig. 5A), and a 2-rate model restricting transitions to the loss of penile sacs (S^+^P^−^→S^−^P^−^) followed by the gain of modified pedipalps (S^−^P^−^→S^−^P^+^) (Fig. 5B). The 2-rate model is an evolutionary trajectory wherein each transition is consistent with escalation in intersexual antagonism during mating. Changes from S^−^P^+^→S^−^P^−^→S^+^P^−^, possible in the 6-rate model, suggest decreasing precopulatory antagonism and/or an increase in reliance on courtship (i.e., female appeasement by the male).

### Evolution of the Penis and Female Genital Operculum

Each species was assigned one of two discrete states for each character. The penis was coded as having either a bilateral pair of cuticular sacs that convey a nuptial gift (S^+^) or as lacking sacs (S^−^); the female genital operculum was coded as either unarmed (B^−^) (Fig. 2B) or as elaborated to form a pregenital barrier (B^+^) (Figs. 2C, 2D). States were determined for all species by original observations of anatomy. We interpreted the evolutionary changes S^+^→S^−^ and/or B^−^→B^+^ as evidence for an increase in precopulatory antagonism and/or a decrease in female appeasement by the male and change in the opposite direction as a decrease in precopulatory antagonism and/or an increase in female appeasement by the male.

Ancestral states were determined for each character using parsimony [39] and a hierarchical Bayesian method [43] implemented in SIMMAP v. 1.5 [44]. In the Bayesian approach, each character was modeled separately in accordance with standards outlined in [45]; we used either an empirical character-bias prior derived from the frequency of terminal states or a β-distribution prior where the best-fit α-shape value was derived from Markov chain Monte Carlo (MCMC) sampling [44]. The overall evolutionary rate for each character set was modeled using a Γ-tree prior obtained via MCMC sampling for the α-shape parameter and β-rate parameter [44]. Analyses were replicated with and without outgroup taxa to assess outgroup effects on the relative rates of character change. Root states were inferred from the marginal posterior probabilities for each state across all sub-sampled, outgroup-rooted trees (n = 431) with fixed branch lengths for each character.

We determined whether state changes in the penis and female genital operculum were correlated using the Discrete module in BayesTraits [40]. This was done by comparing the marginal likelihoods of two models: an independent 4-rate model in which state changes in the penis and female genital operculum were estimated separately (Fig. 5C) and a dependent 8-rate model (Fig. 5D) in which single-step changes between the four penis-operculum combinations (S^+^B^−^, S^+^B^+^, S^−^B^+^, S^−^B^−^) were estimated. Thus a comparison of log likelihoods that favors the 4-rate model indicates no association between state changes, and a comparison that favors the dependent model indicates correlated change between the penis and female genital operculum.

SIMMAP was also used to test for correlations between male and female genital morphology across the posterior tree distribution using predictive sampling and stochastic character mapping via a continuous-time Markov chain [46]. The overall evolutionary rate for each character set was modeled with the Γ-distribution prior used in the ancestral state reconstructions, and bias priors for male and female characters were modeled either as β-distributions or empirical priors as in the ancestral state reconstruction analyses. Bayesian parametric bootstrapping was conducted by sampling each tree 10 times with 10 prior draws for a total of 43,100 samples for all model parameters. Results were summarized as M-values (i.e., the correlation between the histories of two characters across the phylogeny) and p-values (i.e., the probability that an association between penis state and female barrier presence/absence as extreme as observed could arise simply by chance).

In contrast to parsimony, likelihood- or Bayesian-based trait-evolution methods can potentially assess whether change in one state is more likely to precede change in another—even along the same branch—by assigning different rates to these changes. Those states with higher rates are more likely to occur before changes in states with lower rates [47]. Assuming character dependence, it is therefore possible to test whether one character state change (e.g., penis loses sacs) promotes a different character state change (e.g., female gains pregenital barrier). We tested whether nuptial sac loss or pregenital barrier gains were significantly different by using the Discrete module in BayesTraits by comparing a dependent, “precedence-possible” 8-rate model in which transitions between the four penis-operculum combinations were estimated simultaneously (Fig. 5F) to a dependent 7-rate model (Fig. 5E) wherein gain of the pregenital barrier (S^+^B^−^→S^+^B^+^) and loss of penile sacs (S^+^B^−^→S^−^B^−^) were assumed to occur at the same rate. A comparison of log-likelihoods that favors the dependent, “no precedence” 7-rate model would indicate that the rates of increased antagonism from the ancestral condition are equivalent between the sexes, whereas a comparison favoring the 8-rate model indicates a difference between the rates of escalation between the sexes. In the event the 8-rate model is favored, the mean and variance of rates of escalation can be further compared between the sexes. The sex that was most likely to have initiated the escalation can then be determined by its significantly higher mean rate of morphological change.

### General Procedures for BayesTraits Model Testing

All model comparisons in BayesTraits (Fig. 5) were made after analyzing trait evolution using a Markov-chain Monte Carlo algorithm with standard uniform rate priors, 2.1×10^8^ to 6.0×10^9^ iterations, 30% burn-in, and a rate deviation of 0.001–2.0 in order to reach a target acceptance rate of 20–40% per run. At least four independent analyses were performed for each model (see Table 1). Log files were uploaded to TRACER [32] to determine stabilization of log-likelihoods (standard error of no more than 0.03 and a visual inspection of the harmonic mean traces). Although model harmonic means should theoretically approach model marginal model likelihoods [40]–[41]; [48], this use of harmonic means has been criticized [48]–[49]. Therefore we chose to approximate marginal likelihoods using the "Analysis → Calculate Bayes Factors" function of TRACER [33] summarized in [50] with modifications by [51], calculating 1000 bootstrap replicates of the log-likelihood traces. The mean Bayes factor for each model was calculated and used in model log-likelihood comparisons (Table 1). Where model pairs of interest were nested, marginal likelihood approximations were compared using log-likelihood ratio tests. Except where noted, significance was determined where the test statistic value surpassed the χ^2^ distribution critical value at an α value of 0.05. Degrees of freedom were calculated by solving for the difference in estimated parameters.

**Table 1:** Model Bayes factors.

Model	Run 1	Run 2	Run 3	Run 4	Mean
**No Precedence (** **Fig. 5A** **)**	−29.497±0.059	−29.661±0.062	−29.54±0.047	−29.723±0.056	−29.605±0.056
**Penis Precedence (** **Fig. 5B** **)**	−30.967±0.048	−31±0.044	−30.952±0.037	−31.493±0.04	−31.103±0.042
**Fixed Sacculate Root (** **Fig. 5A** **)**	−30.168±0.047	−30.145±0.05	−30.088±0.047	−30.171±0.051	−30.143±0.049
**Independent Change (** **Fig. 5C** **)**	−38.492±0.06	−38.404±0.05	−38.466±0.059	−38.393±0.047	−38.439±0.054
**Dependent, Precedence-Possible Change (** **Fig. 5D, 5F** **)**	−35.232±0.037	−32.852±0.047	−33.153±0.062	−33.175±0.058	−33.603±0.051
**No Precedence (** **Fig. 5E** **)**	−38.989±0.019	−38.552±0.018	−38.586±0.018	−38.16±0.019	−38.571±0.019

Bayes factors from four independent runs per model in BayesTraits [40]–[41] and means used in log-likelihood ratio tests. Bayes factors were calculated using TRACER 1.5 [33] with 1000 replicates of the log-likelihood traces. See Fig. 5 for model design details.

## Results

### Evolution of the Penis and Male Pedipalps

The ancestral male reproductive morphology was inferred by considering the likelihood of the evolutionary trajectory of male traits when root state was fixed or not fixed. A comparison of Bayes factors from the 6-rate, fixed-root model (Fig. 5A), where a root state of S^+^P^−^ was constrained, to those from a similar model where no constraint was imposed, showed no appreciable difference in marginal likelihoods of the models (K = 0.538). This result indicates that the co-occurrence of penile sacs and simple male pedipalps is the primitive state for the eastern North American clade of leiobunine harvestmen, which is consistent with the conclusion based on parsimony (Fig. 4).

Comparison of the marginal likelihood approximations of the 6-rate “no precedence” model, where change between any of the three discrete male reproductive characters is possible, to a 2-rate “penis-precedence” model, where only two transitions are allowed, indicated no significant difference between the two models (log-likelihood ratio test: χ^2^ = 2.996, D.F. = 4, p>0.1). Given this result, the simpler 2-rate “penis-precedence” model is preferred, and we conclude that there may have been a tendency for penile sacs to be lost before the male pedipalps were enhanced for clasping the female. The likelihood of this model is further supported by the lack of species with both enhanced pedipalps and penile sacs.

### Evolution of the Penis and Female Genital Operculum

In order to assess the ancestral states of male and female reproductive morphology with hierarchical Bayesian analysis, the probability distribution priors were estimated for each character set using an MCMC-sampling method [43]. In all SIMMAP analyses, Γ-tree priors for the overall evolutionary rate of each trait were applied. The overall evolutionary rate best-fit shape (α) and rate (β) parameters for penis morphology were α = 3.515, β = 0.038, and for barrier presence, α = 3.108, β = 0.036. Character-bias priors were modeled with either an empirical approach based on the frequency of tip states, or with a β-distribution prior. The best-fit α values for each character-bias distribution were α = 5.888 for penis morphology and α = 5.906 for morphology of the female genital operculum.

Character mapping under parsimony (Fig. 4) supported the parallel loss of penile sacs from a sacculate ancestor (S^+^→S^−^) and gain of female pregenital barricade from ancestors with an unarmed genital operculum (B^−^→B^+^). At least four such transitions are necessary for each character (Fig. 4), although this number is dependent on topology, which we varied in our analyses due to species paraphyly [26]. These results were consistent with those obtained from the Bayesian approach implemented in SIMMAP. The presence of penile sacs (S^+^) was recovered as the ancestral male character state with marginal posterior probabilities ranging from 78% to 80% (with probability being dependent on use of either the two-state empirical or β-bias prior and inclusion/exclusion of the outgroup). Absence of a pregenital barrier (B^−^) was the most likely ancestral female character state, with marginal posterior probabilities of 77% to 96% (with the probability being dependent solely on inclusion or exclusion of outgroup character states in the analysis). Results from two methods thus support an ancestral taxon wherein males had sacculate penes and females lacked a pregenital barrier.

To assess the hypothesis that there are two syndromes of coevolved morphological features, we first needed to determine whether state changes in the penis and female barrier were correlated. We used the BayesTraits Discrete module to compare marginal likelihoods of two alternative hypotheses modeling either independent or dependent change in traits (Figs. 5C vs 5D). Log-likelihood ratio tests of the marginal likelihood approximations of these models favored the dependent, 8-rate model (χ^2^ = 9.672, D.F. = 4, p<0.05). We conclude that the evolution of male and female reproductive structures is correlated across the phylogeny.

Using SIMMAP we also demonstrated a correlation between male and female reproductive morphology. Bayesian predictive distributions were generated using stochastic mapping of male and female reproductive traits to the filtered posterior tree distribution. When compared to the actual trait states by species, a mean correlation between penis morphology and female pregenital barrier presence of 0.147 (p<0.01) was found under the empirical prior, and a correlation of 0.151 (p<0.05) was derived using the β-bias prior. Individual state covariation between sacculate penis type and absent pregenital barrier (Empirical: m_00_ = 0.063, p<0.01, β: m_00_ = 0.065, p<0.01) and non-sacculate penis type and present pregenital barrier (Empirical: m_11_ = 0.068, p<0.01, β: m_11_ = 0.07, p<0.05) was found to be positive and significantly distinct from the predictive distribution. The relationships of sacculate penis type with presence of pregenital barrier (Empirical: m_01_ = −0.054, p<0.01, β: m_01_ = −0.055, p<0.01) and non-sacculate penis type with a lack of female pregenital barrier (Empirical: m_10_ = −0.052, p<0.01, β: m_10_ = −0.053, p<0.01)—both trait combinations seen in a small but non-zero number of species in the phylogeny—co-varied negatively, yet remained significantly different from the predictive distribution.

As male and female morphology was demonstrated to covary across the phylogeny, we additionally tested whether the evolutionary rate at which penile sacs were lost was equal or unequal to the rate at which females acquired the pregenital barrier, all relative to the sacculate, barrier-free ancestor. We compared the likelihood of a 7-rate “No Precedence” model where the rates of pregenital barrier acquisition and loss of penile sacs were forced to be equal, to an 8-rate "Precedence-Possible" model (Fig. 5E). Comparisons of marginal likelihoods revealed a significant difference between models and favored the 8-rate scheme (Fig. 5F) (χ^2^ = 9.936, D.F. = 1, p<0.01). Thus, the rates of change of the penis and female genital operculum cannot be assumed to be equal, and the precedence of one sex's trait change over the other is supported. However when comparing rates of character change and accounting for rate variance, the “Precedence-Possible” model does not appear, on average, to estimate a higher rate for either transition (μ_q12 vs.q13_ = 10.94±18.27, D.F. = 3, t = 1.905, p = 0.0765). An increased number of simulations or alternative priors on μ might change the significance of this difference. Ultimately, there is evidence that changes in the penis and female genital operculum are correlated and that rates of state changes are unequal, which suggests that change in one may precede change in the other. Parsimony on the backbone constraint tree (Fig. 4) suggests pregenital barrier development may have preceded the loss of sacs, but this result is subject to tree topology. As uncertainty in topology was included in the model testing procedure, no conclusion regarding character evolution precedence may be made by parsimony alone, although improved sampling and resolution of the *Hadrobunus* species group might alter this. We found no evidence that change in one sex strongly tended to precede change in the other.

## Discussion

### Patterns in the Evolution of Reproductive Structures in Leiobunine Harvestmen

The results from our analysis indicate that the leiobunine harvestmen of eastern North America are descended from an ancestor with reproductive structures that are consistent with a mating system dominated by courtship where males entice or appease females to obtain copulation. Results from both Bayesian and parsimony-based methods of character reconstruction showed that ancestral males had a subterminal pair of penile cuticular sacs used in conveying a nuptial secretion to the female during the precopulatory phase of mating (Figs. 1B, 3). The male pedipalps were used to clasp the female at the base of her second leg pair but were morphologically similar to those of females. The pregenital openings of female lacked sclerites that might serve as a barrier to forced intromission by the male. This syndrome of reproductive features has persisted in several diverse lineages, and these offer opportunities to explore further the details of the ancestral system. It is reasonable to suppose that the evolutionary mechanism of female mate choice has played a predominant role in shaping the ancestral reproductive syndrome.

There have also been at least four phylogenetically independent transitions from the ancestral system toward morphologies and behaviors consistent with precopulatory antagonistic behavior. This assumes no parallel gains of the nuptial gift sacs, a reasonable assumption given the complexity of the structure and its function in mating [52]. We suspect that the number of independent transitions from sacculate to non-sacculate conditions will increase as phylogenetic relationships within the *Hadrobunus* group are clarified. In each case, penile sacs have been lost and females have evolved sclerotized pregenital barriers. In addition, the male pedipalps of species within the *calcar* and *vittatum* species-groups are enhanced for clasping the female. Each transition has resulted in a different construction of the penis, the female pregenital barrier (Fig. 2) and male pedipalps (compare Figs. 1A, 12).

Our results indicate that loss of penile sacs and elaboration of male pedipalps are correlated. In fact, modified pedipalps always co-occur with non-sacculate penes, although not all non-sacculate species have enhanced male pedipalps. This evolutionary trajectory supports the hypothesis that precopulatory antagonism has originated or increased several times in leiobunine phylogeny, a hypothesis further supported by the correlation found between male and female morphological states. There is some additional evidence from our Bayesian modeling of the evolution of the penis and male pedipalps that the loss of penile sacs tends to precede the elaboration of the male pedipalps (Fig. 4). Interestingly, there are few morphological specializations in females that appear to be dedicated to resisting clasping by males. The only possible exception occurs in the *Hadrobunus* group, where females in all species (both sacculate and non-sacculate) have a spike-like process or “coxal spur” on the posterior margin of the basal segment of the second leg, where the base of the male pedipalpal tarsus likely contacts the female [53].

The timing of the loss of penile sacs and gain of pregenital barriers are strongly correlated. The Bayesian analysis of character evolution showed that a difference likely exists in the rates of the two transformations, which may indicate a tendency for one kind of evolutionary change to precede the other [47]. However, additional tests aimed at resolving these rates failed to find significant differences, and it was not possible to determine whether evolution in the structures of one sex tends to lead the coevolutionary change.

The distribution of morphological characters made parsimony-based character mapping similarly uninformative for reconstructing the sequence of change in the penis and pregenital barrier. The ancestral condition (penile sacs present, pregenital barrier absent) and one derived condition (penile sacs absent, pregenital barrier present) were by far the most common, but unambiguous losses of penile sacs and gains of female pregenital barriers mapped to the same branches and were necessarily interpreted as effectively simultaneous events. However, two species, *Hadrobunus grandis* and an undescribed *Hadrobunus* (*H.* n. sp. 3 IL) have both sacs and barriers, and one species, *Leiobunum relictum*, lacks both sacs and barriers. Depending on their exact phylogenetic positions, these species could represent either an intermediate stage in the transition from courtship to antagonism or a reversal from antagonism back to courtship. Although we regard a reversal to the sacculate condition per se as unlikely [52], the secondary loss of a pregenital barrier is more plausible. In fact, both *L. relictum* and the undescribed *Hadrobunus* n. sp. 2 MO show evidence of incipient or vestigial pregenital barrier structures that are fully developed in closely related taxa. The phylogenetic placement of these species requires corroboration by additional molecular data and analyses.

### Explaining Evolutionary Change in Reproductive Structures

Our present work on the natural history and morphology of leiobunine harvestmen suggests an association between the type of precopulatory mechanism within a species and the duration of its breeding season. Specifically, species with potentially longer breeding seasons tend to have sacculate penes and other features consistent with female enticement by males, while species with shorter breeding seasons tend to have non-sacculate penes and traits associated with precopulatory antagonism. Tropical leiobunines have potentially long breeding seasons and virtually all species retain the ancestral conditions of sacculate penes, simple male pedipalps and unfortified female pregenital openings (J.W. Shultz, pers. obs.). Furthermore, males of these species are typically much smaller than females and tend to have short, poorly sclerotized penes with relatively large gift-bearing sacs. In contrast, species with features consistent with precopulatory antagonism (non-sacculate penes, enlarged male pedipalps, female pregenital barriers) are limited almost exclusively to north temperate regions (J.W. Shultz, pers. obs.), where breeding seasons are presumably limited by the onset of cold winters. Non-sacculate species overwinter as eggs and reach the final instar in mid-summer or later. Significantly, those populations with the most well-developed male palps and female pregenital barriers tend to occur on mountains (e.g., *Leiobunum hoffmani* and *L. calcar*) [54], where breeding seasons are likely to be short. There are also sacculate species in the north temperate region but most overwinter as immatures, attain adulthood in late spring and have potentially long breeding seasons (Fig. 4: "early-season" clade). There are exceptions to these patterns (e.g., *L. aldrichi* and *L. politum* are sacculate but mature in summer), and the precise onset of sexual maturity and duration of breeding seasons are unknown for all species. Additional research will be required to define the precise reproductive phenology for all eastern leiobunines, but these differences may be key to identifying the mechanism(s) by which reproductive structures have diversified. In light of these life history traits, multiple coevolutionary scenarios may be invoked to identify the origin and/or maintenance of reproductive morphology in the leiobunine harvestmen. We offer three hypotheses that may explain the association between male and female armaments observed across the phylogeny.

#### 1. Natural Selection and the Resource-limitation Hypothesis

In primitively sacculate leiobunines, males make a material contribution to females in the form of an apparently all-or-nothing primary nuptial gift delivered by penile sacs as well as a secondary gift offered directly from the male accessory glands. The environment could impact male genitalic structure indirectly via fitness costs associated with the time and energy used in producing nuptial gifts. Long breeding seasons may provide ample time to replenish gifts, and the cost of losing a gift to an unreceptive female may be relatively low. However, short breeding seasons offer less time for males to acquire the raw materials to produce new gifts [55], and wasting gifts on unreceptive females may result in high fitness costs [56]. The effect could be exacerbated if resource limitations also result in females placing greater demands on males for nutritional gifts prior to copulation. In populations where breeding seasons are short, natural selection could favor changes that reduce male costs, such as the reduction or loss of the all-or-nothing primary gift and the penile sacs that them. Predictions of this hypothesis could be tested in sacculate species by comparing mechanisms of gift delivery in populations with breeding seasons of different durations. These tests would require the use of continuously varying features rather than the presence/absence characters examined here.

Reduction or loss of the primary nuptial gift would presumably entail an evolutionary response in mechanisms that govern female receptivity [56], but it seems unlikely to result directly in the evolution of female pregenital barriers; that is, the reduction of nuptial gifts is not in itself a coercive or antagonistic change warranting the evolution of resistance structures in females. However, it may be that a behavioral form of precopulatory antagonism was present as a facultative strategy in the ancestral mating system or was regularly adopted near the end of the breeding season when males no longer had sufficient time to replenish nuptial gifts. Thus, shorter breeding seasons may shift the relative duration and/or intensity of ancestrally coexisting strategies, as seen experimentally in seed beetles [57], and this could explain the coevolutionary loss of penile sacs and gain of female pregenital barriers found in our study system.

Whether or not behavioral precopulatory antagonism existed in the ancestral mating system or evolved later—perhaps in response to environmental effects on males—two additional alternative hypotheses may account for antagonistic morphologies observed in leiobunine harvestmen.

#### 2. Female Choice and the Shifting-signal Hypothesis

The ancestral presence of gift-bearing penile sacs is consistent with a mating system dominated by female mate choice; females may have chosen males based on the quality of their material “display.” If the loss of penile sacs reflects excessive male fitness costs imposed by short breeding seasons, the ancestral material signal would need to be replaced by a different signal if female choice is to persist. The correlated loss of penile sacs and origin of female pregenital barriers may reflect a shift from a nutritional/chemical signal of male quality to a mechanical/stimulatory signal. Coevolution of reproductive “armaments” between the sexes could reflect competition among males to enhance the mechanical signal offered to females (i.e., force produced by the penis or pedipalps) and enhancements to the female that allow her to safely assess forceful mechanical signals (i.e., the female pregenital “barrier”). This evolutionary process might outwardly resemble sexually antagonistic coevolution, but would be maintained as a form of female choice for superior mates by using female "resistance" as a screen [11]; [58]. However, persistent control of mating outcomes by females in this system would require the female to be mechanically superior to males, unless forced copulation itself represents a kind of female choice [59]. One implication of the shifting-signal hypothesis is that the ancestral nutritional/chemical signals appear to be a direct fitness benefit to the female while the mechanical signal represents indirect benefits through increased offspring viability via good genes [60] or the product of a Fisherian sexy sons process [61]–[62]. Evidence from other systems indicates that offspring resulting from coercive encounters may have lower fitness [63]–[64], but the question of whether the indirect benefits derived from female preferences for coercive males are significant enough to drive changes in female resistance has yet to be answered to the satisfaction of the field [59]; [65].

#### 3. Intersexual Conflict and the Male-male Competition Hypothesis

Shortened breeding seasons should increase competition among males for access to females, especially within polygynadrous species like harvestmen. Mechanisms of male-male competition can themselves be detrimental to female fitness, whether by overriding female preferences and preventing females from mating with preferred suitors [66] or by producing structures and behaviors in the context of intrasexual conflict that lead to female loss of fitness during mating [58]. Males may monopolize females via prolonged pedipalpal clasping or mate guarding [67], thereby limiting the time available to the female for feeding, oviposition or mating with preferred males [68], while also exposing the female to predators [69]. While superficially appearing to be beneficial or at least not harmful to females by reducing mating rate [70], these male behaviors may have a net detrimental effect on female fitness. Also, by-products of sperm competition, a form of post-copulatory male-male competition, may lower female long-term fertility [70] or longevity [71].

The hypotheses proposed here invoke an overarching role for the environment in precipitating evolutionary change in reproductive structure and behavior and thereby offer an alternative to the near-exclusive focus on female choice and sexual conflict that have tended to dominate recent discussions. Our proposals anticipate a positive relationship between the duration of breeding season and the intensity of material-based courtship and/or an inverse relationship with the intensity of forceful interactions between the sexes. The focus on duration of breeding season does not deny significant roles to either female choice or sexual conflict in shaping reproductive evolution but offers a testable explanation of reproductive diversity by assessing the strength of associations between ecological, morphological, and behavioral variables. In contrast, the predictions of female choice and sexual conflict tend to differ mainly in the difficult-to-measure fitness outcomes for the two sexes [72]. Indeed, when considering fitness in the broad sense, antagonistic precopulatory behavior appears to be explained as readily by female choice for male mechanical abilities as by intersexual conflict [59]. Progress towards integrating these heretofore competing mechanisms may require an alternative perspective, like the one initiated in this paper.

## Supporting Information

Table S1 **Taxon sampling for BEAST v1.7.1 phylogenetic reconstruction and reproductive trait evaluation.** Accession numbers are for the GenBank sequence repository; numbers GQ870643–GQ870668 and GQ872152–GQ872185 are derived from [74]. Columns 5 and 6 include relevant papers on species morphology [53]–[54]; [75]–[78] and/or numbers of male and female specimens analyzed for current study.
(DOCX)
