# Supplementary material for: Comparative Analyses of Reproductive Structures in Harvestmen (Opiliones) Reveal Multiple Transitions from Courtship to Precopulatory Antagonism
Source: PLoS One. 2013 Jun 10;8(6):e66767. doi: 10.1371/journal.pone.0066767 (PMC3677920; doi:10.1371/journal.pone.0066767)
Supplement: Table S1 — Taxon sampling for BEAST v1.7.1 phylogenetic reconstruction and reproductive trait evaluation. Accession numbers are for the GenBank sequence repository; numbers GQ870643–GQ870668 and GQ872152–GQ872185 are derived from [74]. Columns 5 and 6 include relevant papers on species morphology [53]–[54]; [75]–[78] and/or numbers of male and female specimens analyzed for current study. (DOCX) [file pone.0066767.s001.docx]

| Species | Accession numbers | Sequence specimen locality | Morphological specimen locality | Male traits | Female traits |
| --- | --- | --- | --- | --- | --- |
| Eumesosoma roeweri | JQ432365,  JQ432307, JQ432253 | USA: TX: Wichita Co. | USA: TX: Travis Co., USA: TX: Wichita Co.,  USA: TX:  Williamson Co. | 6 specimens | 5 specimens |
| Hadrobunus grandis | JQ432358, JQ432300, JQ432249 | USA: FL: Alachua Co. | USA: FL: Alachua Co. | 4 specimens; [53] | 5 specimens; [53] |
| Hadrobunus n. sp. 3 IL “hedini” | JQ432363, JQ432305 | USA: IL: Johnson Co. | USA: IL: Johnson Co. | 2 specimens | 2 specimens |
| Hadrobunus n. sp. 1 TN “hoffmani” | JQ432359, JQ432301, JQ432250 | USA: TN: Sevier Co. | USA: TN: Blount Co.,  USA: WV: Monongalia Co. | 6 specimens | 5 specimens |
| Hadrobunus maculosus | JQ432360, JQ432302,  JQ432361, JQ432362, JQ432303, JQ432304, JQ432251 | USA: MD: Howard Co. | USA: MD: Garrett Co. | 6 specimens | 5 specimens |
| Hadrobunus n. sp. 2 MO “milleri” | JQ432364, JQ432306, JQ432252 | USA: MO: Ozark Co. | USA: KS: Douglas Co. | 2 specimens | 6 specimens |
| Leiobunum aldrichi | GQ870650, JQ432342, JQ432284, GQ872154,  GQ870649, JQ432343, JQ432285, GQ872153,  JQ432344, JQ432286, JQ432238 | USA: MI: Calhoun Co. | USA: OH: Stark Co. | 5 specimens;  [75] | 5 specimens;  [75] |
| Leiobunum bimaculatum | JQ432366, JQ432308 | USA: FL: Jackson Co. | USA: VA: Nansemond Co.,  USA: MS: George Co.,  USA: GA: Toombs Co.,  USA: GA: Tyton Co. | 2 specimens | 6 specimens |
| Leiobunum  bracchiolum | JQ432330, JQ432272, JQ432230 | USA: NC: Guilford Co. | USA: MD: Frederick Co.,  USA: MD:  Prince George’s Co. | 2 specimens | 6 specimens |
| Leiobunum calcar | GQ870653, JQ432316, JQ432258, GQ872157,  JQ432317, JQ432259, JQ432223,  JQ432319, JQ432261,  GQ870655, JQ432320, JQ432262, GQ872158,  JQ432318, JQ432260 | USA: MD: Frederick Co. | USA: NC: Madison Co.,  USA: MD: Garrett Co. | 4 specimens; [54] | 8 specimens; [54] |
| Leiobunum crassipalpe | JQ432331, JQ432273,  JQ432332, JQ432274, JQ432231 | USA: MO: Butler Co. | USA: MO: Butler Co. | 2 specimens | 5 specimens |
| Leiobunum euserratipalpe | JQ432321, JQ432263,  GQ870656, JQ432322, JQ432264, GQ872160 | USA: MD: Montgomery Co. | USA: PA: Bucks Co. | 5 specimens; [54] | 7 specimens; [54] |
| Leiobunum flavum | JQ432353, JQ432295, JQ432245 | USA: AR: Garland Co. | USA: AR: Garland Co. | 5 specimens | 3 specimens |
| Leiobunum formosum | JQ432354, JQ432296,  JQ432356, JQ432298, JQ432247,  JQ432355, JQ432297, JQ432246,  JQ432357, JQ432399, JQ432248 | USA: FL: Jackson Co. | USA: FL: Hernando Co.,  USA: VA: Dickerson Co.,  USA: VA: Northampton Co. ,  USA: FL: Liberty Co. | 5 specimens | 4 specimens |
| Leiobunum hoffmani | GQ870654, JQ432315, JQ432257, GQ872159 | USA: VA: Grayson Co. | USA: VA: Grayson Co. | 6 specimens; [54] | 6 specimens; [54] |
| Leiobunum holtae | JQ432345, JQ432287, JQ432239,  JQ432346, JQ432288, JQ432240 | USA: TN: Cumberland Co. | USA: TN: Hamilton Co.,  USA: TN: Van Buren Co. | 2 specimens | 3 specimens |
| Leiobunum n. sp. 1 NE “minutum” | JQ432352, JQ432294 | USA: NE: Lancaster Co. | USA: NE: Lancaster Co. | 5 specimens | 5 specimens |
| Leiobunum nigropalpi | JQ432323, JQ432265, JQ432224,  JQ432324, JQ432266, JQ432225,  JQ432325, JQ432267, JQ432226 | USA: MD: Frederick Co. | USA: MD: Garrett Co. | 6 specimens; [54] | 11 specimens; [54] |
| Leiobunum politum | JQ432326, JQ432268, JQ432227,  JQ432327, JQ432269, JQ432228,  JQ432328, JQ432270, JQ432229,  JQ432329, JQ432271 | USA: AR: Lawrence Co. | USA: MO: Greene Co.,  USA: MO: Butler Co.,  USA: AR: Lafayette Co. | 2 specimens;  [76] | 4 specimens |
| Leiobunum potosum | JQ432370, JQ432312 | MEXICO: Tlaxcala, Ixtacuixtla | MEXICO: Puebla,  MEXICO: Guerrero | 6 specimens | 5 specimens |
| Leiobunum relictum | JQ432340, JQ432341, JQ432282, JQ432283, JQ432237 | USA: OK: Comanche Co. | USA: OK: Comanche Co. | 3 specimens | 6 specimens |
| Leiobunum royali | JQ432367, JQ432309, JQ432254 | MEXICO: Veracruz, Xalapa | MEXICO: Veracruz | 5 specimens | 6 specimens |
| Leiobunum townsendi | JQ432369, JQ432311 | USA: AZ: Cochise Co. | USA: AZ: Cochise Co. | 5 specimens | 5 specimens |
| Leiobunum uxorium | JQ432339, JQ432281, JQ432235,  JQ432338, JQ432280, JQ432236 | USA: VA: Smythe Co. | USA: VA: King George Co.,  USA: PA: Lancaster Co.,  USA: MD: Howard Co.,  USA: PA: Cumberland Co. | 3 specimens; [76] | 8 specimens |
| Leiobunum ventricosum | JQ432348, JQ432290,  JQ432349, JQ432291, JQ432242,  JQ432350, JQ432292, JQ432243 | USA: TN: Blount Co. | USA: TN: Sevier Co.,  USA: TN: Knox Co.,  USA: KY: Whitley Co. | 4 specimens; [77] | 4 specimens |
| Leiobunum verrucosum | JQ432351, JQ432293, JQ432244,  JQ432347, JQ432289, JQ432241 | USA: TN: Cumberland Co. | USA: KY: Whitley Co. | 4 specimens;  [77-78] | 5 specimens |
| Leiobunum vittatum | JQ432333, JQ432275, JQ432232,  GQ870651, JQ432334, JQ432276, GQ872155,  JQ432335, JQ432277, JQ432233,  JQ432336, JQ432278, JQ432234,  GQ870652, JQ432337, JQ432279, GQ872156 | USA: TN: Davidson Co. | USA: MO: Carter Co.,  USA: AR: Greene Co., | 5 specimens; [77] | 4 specimens |
| Leuronychus pacificus | JQ432368, JQ432310, JQ432253 | USA: AZ: Cochise Co. | USA: AZ: Cochise Co.,  USA: CA: San Diego Co.,  USA: CA: Los Angeles Co.,  USA: CA: Orange Co. | 4 specimens | 3 specimens |
| Togwoteeus biceps | JQ432371, JQ432313 | USA: NM: Taos Co. | USA: NV: White Pine Co.,  USA: NM: Taos Co. | 5 specimens | 2 specimens |
|  |  |  |  |  |  |
